# Ethical challenges of clinical trials with a repurposed drug in outbreaks

**DOI:** 10.1007/s11019-023-10140-4

**Published:** 2023-03-07

**Authors:** Katarzyna Klas, Karolina Strzebonska, Marcin Waligora

**Affiliations:** Research Ethics in Medicine Study Group (REMEDY), Faculty of Health Sciences, Jagiellonian University Medical College, Michalowskiego 12, 31-126 Krakow, PL Poland

**Keywords:** Drug repurposing, Clinical trials, Amantadine, COVID-19, Bioethics

## Abstract

Drug repurposing is a strategy of identifying new potential uses for already existing drugs. Many researchers adopted this method to identify treatment or prevention during the COVID-19 pandemic. However, despite the considerable number of repurposed drugs that were evaluated, only some of them were labeled for new indications. In this article, we present the case of amantadine, a drug commonly used in neurology that attracted new attention during the COVID-19 outbreak. This example illustrates some of the ethical challenges associated with the launch of clinical trials to evaluate already approved drugs. In our discussion, we follow the ethics framework for prioritization of COVID-19 clinical trials proposed by Michelle N Meyer and colleagues (2021). We focus on four criteria: social value, scientific validity, feasibility, and consolidation/collaboration. We claim that launching amantadine trials was ethically justified. Although the scientific value was anticipated to be low, unusually, the social value was expected to be high. This was because of significant social interest in the drug. In our view, this strongly supports the need for evidence to justify why the drug should not be prescribed or privately accessed by interested parties. Otherwise, a lack of evidence-based argument could enhance its uncontrolled use. With this paper, we join the discussion on the lessons learned from the pandemic. Our findings will help to improve future efforts to decide on the launch of clinical trials on approved drugs when dealing with the widespread off-label use of the drug.

## Introduction

Drug repurposing, also known as drug repositioning or drug re-profiling, is a strategy of identifying new potential uses for existing drugs (Pushpakom et al. 2019; Parvathaneni et al. 2019; Ashburn and Thor 2004). Researchers can apply this method at any stage of the drug development process: from a substance at the preclinical level to the drug with authorization approval (Begley et al. 2021). Compared to the classical drug development pathway starting from the discovery of novel compounds, drug repurposing may accelerate the research process. This is because data collected for previously evaluated indications can be used as a basis for research on new efficacy assessment. Consequently, during repurposing some of the initial steps of drug development can be bypassed (Parvathaneni et al. 2019; Ashburn and Thor 2004).

The global outbreak of COVID-19 created unprecedented pressure on clinical trial regulators, ethics committees, researchers, and other stakeholders to develop prevention and treatment as quickly as possible. Various methods were adopted to speed up the delivery of data and verify the research hypothesis (Park et al. 2021). Many researchers turned to the existing drugs, which made drug repurposing a widely applied approach (Sahoo et al. 2021; Riva et al. 2020; Bakowski et al. 2021; Galindez et al. 2021; Venkatesan 2021). However, the urge to do “something” to help save patients with COVID-19 led to many poorly planned actions (Lynch et al. 2021). Many clinical trials testing the same drugs were conducted almost simultaneously, raising concerns about redundancy and waste (Lynch et al. 2021; Meyer et al. 2021; Hutchinson, Klas, Carlisle, Kimmelman, et al. 2022; Maziarz and Stencel 2022). These challenges reinforced the role of informative and ethical clinical research (Meyer et al. 2021; Hutchinson, Klas, Carlisle, Kimmelman, et al. 2022; London and Kimmelman 2020). Numerous guidelines and ethical frameworks were proposed to prevent research waste or misconduct and support clinical research during the COVID-19 pandemic (Hsu et al. 2021; London and Kimmelman 2020; Meyer et al. 2021).

This paper presents the case study of amantadine repurposing during the COVID-19 pandemic. This example illustrates some of the ethical challenges associated with the launch of clinical trials to evaluate already approved drugs. We discuss whether clinical trials of amantadine during the COVID-19 pandemic were ethically justified and should have been initiated. We focus on the impact of high social pressure on rapid clinical testing and the need for evidence, as it was during the COVID-19 pandemic. In our analysis, we follow the ethical framework proposed by Michelle N Meyer and colleagues (2021). We chose this framework as it provides guidance for research institutions on how to coordinate and prioritize research activities in a limited amount of human and material resources (Meyer et al. 2021). It helps to determine clinical trials that are robust enough and feasible to start in the context of specific circumstances triggered by the pandemic.

## Amantadine case study

Amantadine has been used in clinical practice since the 1960s. It was originally developed as a prophylactic agent against influenza. Nowadays, amantadine is more commonly known as an antiparkinsonian agent or a therapy for multiple sclerosis–related fatigue (Nisar et al. 2019; UpToDate 2022). Amantadine is approved in many European countries and in the United States. In the face of the COVID-19 pandemic, amantadine joined a long list of medications considered as possible cures for COVID-19. The drug attracted particular attention in Poland.

One of the first reports on the potential efficacy of adamantanes, a group of chemical compounds that includes amantadine and memantine, in the fight against COVID-19 was published in early 2020 (Rejdak and Grieb 2020). The study was a case series analysis of 22 patients who suffered from Parkinson’s disease, multiple sclerosis, or cognitive impairment and had confirmed SARS-CoV-2 infections. Those patients took amantadine or memantine as part of the regular treatment of neurological disorders. The authors of the study noted that none of the patients developed clinical manifestations of infectious disease, indicating a potential protective role of adamantanes (Rejdak and Grieb 2020).

In October 2020, one of the European clinics announced on its website that amantadine could treat COVID-19 in 48 h (Optima Outpatient Clinic 2020). The author of the post was a physician from Poland. He shared clinical experience on how the drug Viregyt K (Egis Pharmaceuticals PLC) containing amantadine had helped him and his patients recover from COVID-19. The doctor described a detailed therapeutic scheme he created (Optima Outpatient Clinic 2020). Despite the lack of reliable scientific evidence to prove the hypothesis about amantadine’s efficacy, the news was enough to attract not only patient but also healthcare professional attention. In October and November 2020, the number of new cases of COVID-19 significantly increased in Poland (World Health Organization 2022b). Vaccines against COVID-19 were not available at that time. Simultaneously, limited therapeutic options with confirmed activity against COVID-19 were available. Therefore, the information on a new drug that could treat COVID-19 triggered an explosion of public discussion. Soon other anecdotal evidence about the role of amantadine in the treatment of COVID-19 emerged (Wojtasiński 2020; Notes from Poland 2021). No results from clinical trials and only low-quality evidence based on observational (Rejdak and Grieb 2020; Aranda-Abreu et al. 2020, 2021; Mancilla-Galindo et al. 2020; Borra 2020) and preclinical (Smieszek et al. 2020; Abreu et al. 2020) analyses for amantadine use were available. However, the interest in amantadine was tremendous, leading to a substantial increase in the Viregyt K sales in Poland. In October 2020, the sales rose more than three times (from 5000 to 17,000 packages/month) (Ministry of Health 2021).

The widespread off-label use of Viregyt K quickly caused problems with the drug availability on the national level. Patients faced drug shortages. This trend continued even after the health authorities in Poland issued a negative recommendation for amantadine-based COVID-19 treatment (Agency for Health Technology Assessment and Tariff System 2020). The recommendation was based on the results from an observational retrospective study (Mancilla-Galindo et al. 2020) and two case series analyses (Aranda-Abreu et al. 2020; Rejdak and Grieb 2020). Health authorities emphasized that due to the limited scientific evidence and its low reliability, there was a high degree of uncertainty in concluding the efficacy and safety profile of amantadine for COVID-19 treatment. Therefore, in December 2020 the Ministry of Health in Poland imposed a restriction on the sale of Viregyt K. The aim was to ensure access to regular therapy for patients with neurological disorders and to limit the purchase for COVID-19 (Ministry of Health 2020).

In response to the growing interest in the drug and the risk of its widespread use despite the substantial evidentiary gap, the public body, the Medical Research Agency in Poland, funded two non-commercial clinical trials that began in March 2021 (Medical Research Agency in Poland 2021). The goal was to provide evidence and dispel public concerns about the role of amantadine in COVID-19. One trial (TITAN, NCT04952519) evaluated the efficacy of amantadine in the treatment of hospitalized patients with moderate or severe COVID-19 (ClinicalTrials.gov 2021b). The other (COV-PREVENT, NCT04854759) investigated the efficacy of the drug in the initial phase of COVID-19 with less severe symptoms including patients in an ambulatory setting (ClinicalTrials.gov 2021c). The characteristics of the COV-PREVENT and the TITAN trial design are presented in Table 1. The table reports the information presented in two clinical trial registries: ClinicalTrials.gov and The European Union Clinical Trials Register (EU CTR). It also contains data about the third clinical trial (ACT, NCT04894617) that we found in these registries when searching for studies evaluating amantadine in COVID-19. This study was initiated in Denmark (ClinicalTrials.gov 2021a).

**Table 1:** Characteristics of COV-PREVENT, TITAN and ACT clinical trials that evaluated amantadine as COVID-19 treatment

Acronym of the trial	COV-PREVENT	TITAN	ACT
Trial title	The Use of Amantadine in the Prevention of Progression and Treatment of COVID-19 Symptoms in Patients Infected With the SARS-CoV-2 Virus	Efficacy of Amantadine Treatment in COVID-19 Patients	Amantadine for COVID-19: A Randomized, Placebo Controlled, Double-blinded, Clinical Trial
NCT ID	NCT04854759	NCT04952519	NCT04894617
EudraCT Number	2021-001144-98	2021-000981-13	2021-001177-22
Trial Design	- Phase 3 - Randomized - Double-blind	- Phase 3 - Randomized - Double-blind	- Phase 3 - Randomized - Double-blind
Trial participants	Adult hospitalized and non-hospitalized patients in the early phase of COVID-19 who are at the risk for severe illness	Adult hospitalized patients with moderate or severe COVID-19 in the initial stage of the disease	Non-hospitalized high-risk patients with COVID-19
Intervention	Amantadine	Amantadine	Amantadine
Comparator	Placebo	Placebo	Placebo
Primary outcomes	Clinical deterioration	Time to recovery	Clinical status
Location of trial	Poland	Poland	Denmark
Anticipated number of trial centers	8 centers	20 centers	1 center
Estimated Enrollment	200 participants	500 participants	Unclear – it differs between ClinicalTrials.gov (226 participants) and the European Union Clinical Trials Register (242 participants)
Start Date	Unclear - it differs between ClinicalTrials.gov (March 15, 2021) and the European Union Clinical Trials Register (April 20, 2021)	March 30, 2021	Unclear - it differs between ClinicalTrials.gov (June 1, 2021) and the European Union Clinical Trials Register (April 26, 2021)

Interest in amantadine continued throughout 2021 and attracted more attention around the world. More preclinical (Fink et al. 2021; Zhou et al. 2021; Toft-Bertelsen et al. 2021a) and observational data (Kamel et al. 2021; Bodnar et al. 2021) emerged. In one study the authors even proposed amantadine as a ‘novel, cheap, readily available and effective way to treat COVID-19’ (Toft-Bertelsen et al. 2021a). However, this recommendation was based on the results of *in vitro* experiments. As this led to exacerbated misinformation about the drug efficacy, the authors promptly reported a correction to the article (Toft-Bertelsen et al. 2021b).

In February 2022, the preliminary results of the TITAN trial were published in a press release. Data from 149 hospitalized patients (78 received amantadine, 71 received a placebo) were analyzed. No significant differences in efficacy between placebo and amantadine were observed (Notes from Poland 2022). The principal investigator announced the termination of the trial. To our knowledge, as of November 2022, the other two trials (COV-PREVENT and ACT) have not published the trial results yet.

## Ethical challenges

The case of amantadine presents an example of an approved drug that attracted attention as a possible treatment or prevention for COVID-19 leading to the launch of clinical trials. According to Meyer and colleagues (2021), the assessment of the legitimacy of the launch of clinical trials should include three consecutive stages. The initial stage should start with the evaluation of four threshold criteria: (i) social value, (ii) scientific validity, (iii) feasibility and (iv) consolidation/collaboration. The second stage should include the assessment of whether an institution has enough resources to support all trials that fulfill the first stage. If yes – all trials can proceed. If not – the third stage of trials’ evaluation is required, which includes additional criteria for prioritization together with study-specific criteria (e.g., promising intervention or institutional expertise) or institution’s portfolio diversity criteria. In our analysis of amantadine clinical trials, we focus on the requirements of the first stage (Meyer et al. 2021). Without fulfilling these criteria, no clinical trial should be initiated.

### Social value

Social value is one of the fundamental ethical requirements for clinical trials (Wendler and Rid 2017). According to the Council for International Organizations of Medical Sciences (CIOMS) guidelines, it is defined as ‘the importance of information that a study is likely to produce’ (CIOMS 2016). Clinical research has social value if it contributes to useful knowledge that is expected to promote patient or society health. Although amantadine did not seem more promising than the other COVID-19 drug candidates, there are at least a few reasons why it was important to start clinical trials evaluating this drug. We discuss them in detail in the following sections.

#### Widespread off-label use of unproven intervention

Amantadine as a treatment for COVID-19 was used outside of the approved indications, which is widely known as off-label use. This is a common strategy in routine clinical practice, particularly in pediatrics (Hoon et al. 2019) and oncology (Saiyed et al. 2017). However, health professionals before prescribing off-label drugs should evaluate the risk-benefit ratio and clinical appropriateness. Their decisions should be supported by solid scientific evidence (Eguale et al. 2016; Maziarz and Stencel 2022). Public health emergencies such as the COVID-19 pandemic, pose a challenge to the medical environment. Effective treatments are urgently needed, whereas gathering evidence takes time. Simultaneously, the initially obtained evidence is regularly confronted with the results of ongoing clinical trials. Although some intervention appears initially promising, the emerging evidence may fail to support it or even indicate a harmful effect. Hence, the use of unproven interventions should be properly regulated and applied with caution. Their usage should comply with an adequate justification, ethical and regulatory oversight, consent process and contribution to evidence (World Health Organization 2022a). In the case of amantadine, the recommendations of the national health authorities did not support its use as COVID-19 treatment due to the limited scientific evidence and its low reliability (Agency for Health Technology Assessment and Tariff System 2020). In view of this, the regular use of unproven intervention should not be justified. Nevertheless, huge public interest in amantadine contributed to a surge in prescriptions. Many doctors prescribed amantadine in response to patient demand (Notes from Poland 2022, 2021). This mechanism was described by the others as “panic prescribing” (Caplan and Upshur 2020). The provision of reliable evidence from clinical trials was very important to support robust clinical decision-making.

#### Patient safety and uncontrolled use

The high demand for amantadine affected the allocation of the drug. Drug shortage could be a burden for non-COVID-19 patients who needed and benefited from amantadine treatment with confirmed efficacy. To prevent this, Viregyt K sales restrictions were introduced (Ministry of Health 2020). However, this intensified the tensions. Patients tried to obtain the drug on their own (Notes from Poland 2021). Uncontrolled use and self-medication with the drug could pose a real threat to the health and safety of patients. Taking amantadine can result in various side effects. Cardiovascular disorders (e.g. orthostatic hypotension, syncope, peripheral edema) or central nervous disorders (e.g. dizziness, delusions, illusions, hallucinations, paranoia) are very common (UpToDate 2022). On the one hand, it is understandable that patients facing a life-threatening disease are looking for any possible solutions that could help. On the other hand, such action should be prevented. To do so, adequate evidence is required (Caulfield et al. 2021). Results from robust clinical trials would be a strong argument in discussions aimed at minimizing the risk of uncontrolled use of amantadine.

### Scientific validity

Scientific validity is an ethical requirement applying to study design and methodology. All research must be well-planned and conducted in a rigorous manner to produce reliable and valid results (Bernabe, van Thiel, and van Delden 216). Pandemic circumstances are challenging for the research environment. Time for gaining evidence and learning is very tight. However, urgent need for an effective therapeutic and speed in collecting the evidence should not justify lowering scientific standards (London and Kimmelman 2020). The following section analyses the scientific validity of research hypotheses and research design associated with amantadine clinical trials.

#### Research hypothesis

The research hypotheses in clinical trials should be supported by well-grounded evidence and address an important and unresolved question (Zarin, Goodman, and Kimmelman 2019). The choice to test a particular therapeutic target in clinical trials should be well considered and justified, not random. Depending on the phase of drug development, supporting evidence may be related to the pre-clinical or earlier clinical trial results. If the candidate for a new clinical trial is approved, it can also involve results from other post-marketing trials. Firstly, preliminary evidence allows us to assess the risks and benefits ratio for future participants. Secondly, if we decide to verify a particular research hypothesis, we invest human, financial and infrastructural resources. These resources are usually limited. Investing in one of the proposed hypotheses can affect the resources remaining to verify the others (Lynch et al. 2021; Meyer et al. 2021).

Amantadine clinical trials in Poland were initiated mainly in response to social pressure, increased public interest, and media coverage. We assert that the scientific grounds for starting amantadine clinical trials were low quality evidence. Although the scientific value was anticipated to be low, unusually the social value was expected to be high. This was because of significant social interest in the drug. In our view, this strongly supports the need for evidence to justify why amantadine should not be prescribed or privately accessed by interested parties. Otherwise, a lack of evidence-based argument could enhance uncontrolled use of the drug.

#### Study design

Clinical trials should be designed in a way to provide a meaningful research answer and have sufficient power to detect the positive or negative effects of an evaluated intervention (Zarin, Goodman, and Kimmelman 2019). Considering that, randomized controlled at least double-blinded clinical trials are recommended (Hariton and Locascio 2018; London and Kimmelman 2020; Waligora and Klas 2022). These design requirements were fulfilled by amantadine clinical trials. Moreover, as amantadine has been used in clinical practice for many years, clinical trials started directly from phase 3. This is a very common strategy when testing an already authorized medicine (Verbaanderd et al. 2021). However, flexible research designs could be considered in this case (Park et al. 2021; Vanderbeek et al. 2022). For example, instead of launching new clinical trials, amantadine could be evaluated as one of the arms in already ongoing platform clinical trials. Platform clinical trials enable testing of multiple interventions (or therapeutic schedules) under a single study; they allow arms to be added or dropped during the trial course according to the pre-specified criteria and results of interim analysis (Berry, Connor, & Lewis, 2015). The analysis performed by Vanderbeek et al. (2022) indicates that it is a promising solution for rapid evidence generation. Nevertheless, the inclusion of amantadine in other platform studies would require coordinated collaboration with the other bodies. This could make the process of evaluating the amantadine more complicated, especially since the interest in the drug was mainly national. It is difficult to assess how quickly amantadine could be included as an arm in platform trials that were conducted around the world. In our opinion, choosing the model of randomized, double-blinded clinical trial was appropriate.

### Feasibility

Feasibility assessment is a process to determine the possibility of conducting clinical trials from the point of view of the available resources. The aim is to ensure that there is a reasonable probability of achieving the recruitment goals and to answer the research questions. Clinical trials should be launched only if there is sufficient human factor (including patient-participant or research staff availability) and non-human resources (including funding, drug supplies or available infrastructures) to carry out the research (Meyer et al. 2021; Rajadhyaksha 2010).

The COVID-19 pandemic substantially emphasized problems in the conduct and feasibility of clinical trials (Park et al. 2021; Hutchinson, Klas, Carlisle, Kimmelman, et al. 2022; Hutchinson et al. 2022; Lasch et al. 2022; Mitchell et al. 2020). The large number of COVID-19 clinical trials that were launched simultaneously forced competition for resources (the availability of which was already limited due to the heavy burden on the health care system and the large number of COVID-19 patients). As knowledge and understanding of the disease evolved, and as evidence from other studies emerged, some of the research hypotheses were disproven over time. Furthermore, the forecasting of the pandemic dynamics was burdened with a large dose of uncertainty (Ioannidis et al. 2022). Due to the changing nature of the pandemic, the surge and fall in COVID-19 cases, some of the studies were unable to meet their enrollment targets within the specified time frame (Janiaud et al. 2021). Research environments also faced the challenge of ensuring the safety of the clinical trials’ operational staff and patients. To increase feasibility, solutions such as remote monitoring of a clinical trial or even remote efficacy and safety assessment of the treatment and home delivery of investigational medicinal products have become popular, if the patients enrolled did not require hospitalization (Leyens, Simkins, and Horst 2022).

When amantadine clinical trials were launched, most of these aspects were already known. The feasibility criterion for the TITAN and COV-PREVENT trials seems to be met. Firstly, for the implementation of research tasks, financial support was provided from the resources of the Medical Research Agency in Poland. Thanks to that, the trial funding was secured. Secondly, both studies in Poland were multicenter. These increase the possibility of achieving the recruitment goals and allocating the necessary resources.

### Consolidation and collaboration

Research consolidation and collaboration are broad terms that encompass many aspects of the conduct of clinical trials (Meyer et al. 2021).

First, this requirement deals with cooperation between various institutions involved, including research centers, public-private partnership, and the support provided by regulators. It should be reflected at both regional and international level. The aim is to avoid research waste and maximize the feasibility and efficacy of actions taken (Meyer et al. 2021; Kim and Hasford 2020). In the light of this requirement, the fact that two separate clinical trials evaluating the efficacy of amantadine were launched almost simultaneously in the same country may be questioned. Although the trials tested the efficacy of amantadine in different phases of COVID-19, they could compete for recruitment targets. Furthermore, shortly after the simultaneous start of two clinical trials, another trial in Denmark began to evaluate the efficacy of amantadine in COVID-19. This raises questions about the lack of coordination of research activities at the international level. Given the limited scientific values of the research hypothesis, the number of study participants potentially exposed to this drug should be reduced.

Second, rapid and transparent data sharing contributes to the consolidation of research efforts (Naci et al. 2020; Strzebonska et al. 2020). This applies not only to the sharing of results, but also to research protocols. Especially during a health crisis, it is important to exchange information as quickly as possible. The quality of the reporting varies between amantadine clinical trials conducted in Poland and Denmark. For example, on ClinicalTrials.gov the description of clinical trials conducted in Poland is limited to one or two sentences (ClinicalTrials.gov 2021b, c), while the description of the clinical trial conducted in Denmark is more detailed and comprehensive (ClinicalTrials.gov 2021a). There is also inconsistency between information reported for the trials in the ClinicalTrials.gov and the European Union Clinical Trials Register (EU CTR). The description of TITAN trials seems to be more detailed in EU CTR (e.g. it includes data about secondary endpoints, whereas ClinicalTrials.gov does not), but most data on EU CTR are in the Polish language, which limits its availability (ClinicalTrials.gov 2021b; The EU Clinical Trials Register 2021b). In the case of ACT, the planned number of patients included in the study is different in each registry (at ClinicalTrials.gov there is a number of 226 patients (ClinicalTrials.gov 2021a), whereas at EU CTR – 242 (The EU Clinical Trials Register 2021a)). Importantly, for now, we can learn only from the press materials that in the TITAN study, a drug called remdesivir was used as a standard of care in the experimental and placebo group. It is unclear whether other drugs were also used. Lack of trial transparency disturb the assessment of the credibility and quality of the study for interested parties (e.g., potential participants, other researchers).

## Limitations

The following limitations should be considered in our analysis. We built our arguments based on publicly available data. We did not have access to full research protocols and other internal documents shared between the research sponsor and the bioethics committee or the national competent authority. Finally, our article emphasized amantadine trials launched in Poland. We have little information about the study in Denmark. Therefore, we could not accurately describe and compare the situation in both countries.

## Conclusion

In this paper, we analyzed the case study of amantadine clinical trials. Despite the lack of high-quality evidence that could prove the drug’s efficacy, these clinical trials were launched in response to social demand resulting from the widespread and uncontrolled use of amantadine outside the label. We aimed to discuss whether amantadine clinical trials should have been conducted. To do so, we followed four ethical aspects proposed as threshold criteria for prioritization of COVID-19 clinical trials: social value, scientific validity, feasibility, and consolidation/collaboration (Meyer et al. 2021). Our analysis indicates doubts regarding the fulfillment of individual criteria, especially in terms of scientific validity and quality of evidence supporting the research hypothesis. However, although the scientific value was low, the social value of amantadine clinical trials was extremely high. Launching clinical trials was necessary to gain solid evidence verifying amantadine efficacy. This was important to support the clinical decision-making process while reassuring public concerns. With our article, we join the discussion on the lessons learned from the pandemic. Our findings may help to improve future efforts to decide on the launch of clinical trials on approved drugs when dealing with the widespread off-label use of the drug.

## Data Availability

The data used in this article are from publications available in the public domain.
